# ASK1 Enhances Angiotensin II-Induced Liver Fibrosis *In Vitro* by Mediating Endoplasmic Reticulum Stress-Dependent Exosomes

**DOI:** 10.1155/2020/8183713

**Published:** 2020-11-07

**Authors:** Pei-pei Fang, Chen-wei Pan, Wei Lin, Jie Li, Shan-shan Huang, Guang-yao Zhou, Wen-jun Du, Qiang Li

**Affiliations:** ^1^Division of Liver Diseases, Jinan Infectious Disease Hospital, Shandong University, China; ^2^Department of Infectious Disease, The Second Affiliated Hospital and Yuying Children's Hospital of Wenzhou Medical University, 325000, China

## Abstract

**Background:**

Apoptosis signal-regulating kinase 1 (ASK1) has been reported to induce fibrotic signaling in the setting of oxidative stress. However, the role of ASK1 and its mechanism of action in angiotensin II- (Ang II-) induced liver fibrosis remain largely unknown.

**Methods:**

Human hepatic LX-2 stellate cells were treated with Ang II alone or cotreated with Ang II plus an ASK1 inhibitor (GS-4997) or siRNA-targeting ASK1. Immunofluorescent staining, real-time PCR, and western blotting were used to determine the expressionof *α*-SMA, Col I, and Col III expression. Cell viability was assessed by the CCK-8 assay. The concentrations of IL-1*β*, IL-18, and TNF-*α* in conditioned medium were determined by ELISA. The levels of intracellular ROS in LX-2 cells were analyzed using a ROS assay kit. Exosome size was determined by electron microscopy.

**Results:**

Ang II markedly increased the expression of extracellular matrix (ECM) proteins (*α*-SMA, Col I, and Col III) and proinflammatory cytokines (IL-1*β*, IL-18, and TNF-*α*). Ang II also increased the expression of endoplasmic reticulum stress (ERS) markers (GRP78, p-PERK, and CHOP) and p-ASK1. Results also showed that pretreatment with GS-4997 or siRNA could abolish all the abovementioned effects on LX-2 cells. Furthermore, we found that exosome release caused by ASK1-mediated ERS was involved in the activation of LX-2 cells by Ang II. The activation of LX-2 cells could be blocked by treating the exosomes with annexin.

**Conclusions:**

In summary, we found that ASK1 mediates Ang II-activated ERS in HSCs and the subsequent activation of HSCs, suggesting a promising strategy for treating liver fibrosis.

## 1. Introduction

Liver fibrosis is a common form of chronic liver disease that may progress to liver cirrhosis if not controlled [1]. As the predominant type of mesenchymal cell in the liver, hepatic stellate cells (HSCs) play a critical role in the process of liver fibrosis. While HSCs are usually quiescent under normal physiological conditions [2], they are capable of responding to an external stimulus that transforms them into myofibroblast-like cells characterized by increased proliferation and excessive accumulations of *α*-smooth muscle actin (*α*-SMA) and collagen type I (Col-1) after being activated [3]. Liver transplantation is the most effective treatment used to treat patients with liver fibrosis; however, the limited supply of organ donors often makes that treatment unavailable and unpopular [4]. Therefore, it is important to investigate the mechanisms underlying liver fibrosis and HSC activation in order to develop new methods of treatment.

Extracellular vesicles, including exosomes, microvesicles, and apoptotic bodies, have been regarded as key regulators in information transduction [5]. Exosomes are small particles with a diameter of 40 to 120 nm and contain miRNAs, noncoding RNAs, and proteins that facilitate intercellular communication [6]. Basically, exosomes can be released from a wide variety of cells [5]. Mesenchymal stem cell-derived exosomes have been considered for use as therapeutic agents for treating liver diseases, including liver fibrosis [7]. Rong et al. found that human bone mesenchymal cell-derived exosomes (hBM-MSCs-Ex) could ameliorate liver fibrosis by attenuating HSC activation via the Wnt/*β*-catenin pathway [8]. A recent study showed that exosomes secreted from HSCs contribute to liver fibrosis by delivering glycolysis-related proteins that subsequently alter cell metabolism [9]. This finding suggests that activated HSCs might be capable of initiating liver fibrosis.

Accumulating evidence suggests that the renin-angiotensin system (RAS), HSC activation, and hepatic fibrogenesis are closely linked [10–12]. As the main effector of RAS, angiotensin II (Ang II) has been shown to promote HSC proliferation, increase ECM protein expression, and induce oxidative stress and hepatic inflammation [13, 14]. Many studies have reported that inhibition of Ang II or a blockade of Ang II receptor type I (AT1R) can attenuate liver fibrosis by reducing HSC activation and ECM synthesis [15–18]. Another study suggested that inhibition of Ang II intracellular signaling pathways might prevent the progression of liver fibrosis [19]. Among those signaling pathways, pathways involving oxidative stress, transcription factor nuclear factor *κ*B (NF-*κ*B), and especially signaling molecules called mitogen-activated protein kinases (MAPKs) have received great attention due to their important functions in the pathogenesis of fibrosis, including liver fibrosis [20]. In addition, *in vivo* and *in vitro* studies have shown that enrichment of Ang II leads to activation of endoplasmic reticulum stress (ERS) in cardiomyocytes [21]. However, it remains unclear whether Ang II is responsible for the ERS that occurs in liver fibrosis.

Apoptosis signal-regulating kinase 1 (ASK1) is a member of the MAPK kinase kinase (MAP3Ks) family, which has been reported to be an upstream activator of the c-Jun N-terminal kinase (JNK) and p38 MAPK signaling cascades [22, 23]. Various stressors, including ERS, reactive oxygen species (ROS), and lipopolysaccharides (LPS), can phosphorylate ASK1 to subsequently induce the activation of JNK and p38 MAPK and thus participate in modulating cell proliferation, apoptosis, and inflammation [24–26]. Yamada et al. [27] reported that phosphorylated ASK1 was expressed in atherosclerotic lesions and liver tissues with bile-duct-ligation- (BDL-) induced injuries. Moreover, Noguchi et al. [28] suggested that use of a specific ASK1 pathway blocker or inhibitor might be a useful strategy for treating human cholestatic diseases by demonstrating that ASK1-mediated hepatic necroinflammation and proliferation are closely linked to liver fibrosis and fibrogenesis. Nevertheless, the potential link between ASK1 and ERS during Ang II-induced HSC activation remains unclear and requires further investigation.

In view of the possible role played by ASK1 in liver fibrogenesis, we performed *in vitro* experiments designed to elucidate the effects of ASK1 on Ang II-induced HSC proliferation, ECM-related protein expression, ERS, and inflammation to gain a better understanding of the roles played by ASK1 and Ang II in liver fibrosis.

## 2. Materials and Methods

### 2.1. Main Materials and Reagents

Human angiotensin II (Ang II), GS-4997 (an ASK1 inhibitor), and bicinchoninic acid (BCA) assay kits were purchased from Sigma-Aldrich (St. Louis, MO, USA). Primary antibodies against the following proteins were used: *α*-smooth muscle actin (*α*-SMA), apoptosis signal-regulating kinase 1 (ASK1), p-ASK1, collagen type I (Col I), and Col III (Cell Signaling Technology Inc., Beverly, MA, USA); GRP78, PERK, p-PERK, and CHOP (Abcam, Cambridge, MA, USA); and CD63, TSG, and GAPDH (Santa Cruz Biotechnology Inc., Dallas TX, USA). A fluorescein isothiocyanate- (FITC-) conjugated horseradish peroxidase- (HRP-) conjugated secondary antibody and an enhanced chemiluminescence kit were obtained from the Beyotime Institute of Biotechnology (Haimen, Jiangsu, China). Dulbecco's modified Eagle's medium (DMEM) and fetal bovine serum (FBS) were obtained from Gibco (Carlsbad, CA, USA). A reactive oxygen species (ROS) assay kit was purchased from Beijing Solarbio Science & Technology Co., Ltd.; TRIzol reagent was purchased from Invitrogen (Carlsbad, CA, USA); and Power SYBR Green PCR Master Mix was purchased from Applied Biosystems (Foster City, CA, USA).

### 2.2. Cell Culture, Groups, and Transfection

The immortalized human hepatic stellate cell (HSC) line LX-2 was purchased from Meixuan Biological Science and Technology Ltd. (Shanghai, China) and routinely cultured in DMEM supplemented with 10% FBS at 37°C in a 95% air/5% CO_2_ humidified atmosphere. For the *in vitro* experiments, LX-2 cells were divided into the following three groups: (1) blank group: only DMEM medium was added, (2) Ang II group: LX-2 cells were treated with Ang II (10^−7^ M) for 4 h, and (3) Ang II+GS-4997 group: LX-2 cells were treated with Ang II plus GS-4997 for 4 h.

siRNA (5′-GGUCGAAUCUACAAAGAUATT-3′) and NC (5′-GAACGACUAGUCAAUGAUATT-3′) were purchased by GenePharma (Shanghai, China). Transfections were performed by using Lipofectamine 2000 according to the manufacturer's instructions.

### 2.3. Immunofluorescence Staining

Briefly, LX-2 cells were harvested and fixed with 4% paraformaldehyde at room temperature for 15 min and then permeabilized with 0.5% Triton X-100 (Invitrogen) for 10 min. After washing with PBS, the cells were incubated overnight with a primary antibody against *α*-SMA at 4°C and then stained with the FITC-labeled secondary antibody. The cell nucleus was visualized by staining with 4′,6-diamidino-2-phenylindole (DAPI). Subsequently, the stained cells were observed under a fluorescence microscope (Leica, Germany).

### 2.4. Cell Viability Assay

Cell viability was determined by using the Cell Counting Kit-8 (CCK-8) assay. Approximately 1 × 10^4^ LX-2 cells were seeded into each well of triplicate 96-well culture plates and cultured for 24, 48, and 72 h, respectively. Next, 10 *μ*L of CCK-8 reagent solution (Dojindo, Kumamoto, Japan) was added to each well, and the cells were incubated for an additional 2 h at 37°C. Cell viability was evaluated by measuring the optical density (OD) of each well at 450 nm with a microplate reader.

### 2.5. Quantitative Real-Time PCR

Total RNA was isolated from LX-2 cells using the TRIzol reagent and then reverse-transcribed into cDNA by using a first-strand cDNA synthesis kit (TOYOBO, Osaka, Japan) according to the manufacturer's protocol. Quantitative real-time PCR was subsequently performed by using the Power SYBR Green PCR Master Mix, and the primer sequences used in this study are listed in Table 1. The relative levels of gene expression were determined using the 2^−*ΔΔ*Ct^ method [29] with *GAPDH* serving as an internal reference gene.

### 2.6. Western Blotting

LX-2 cells were rinsed twice with ice-cold PBS and then lysed in RIPA lysis buffer (Sigma-Aldrich). The lysed cells were centrifuged at 10,000 g for 15 min at 4°C, and the supernatants were harvested. The protein concentration of each supernatant fraction was determined using a BCA kit. Equal amounts of protein (40 *μ*g) were separated by 12% SDS-PAGE, and the protein bands were transferred onto polyvinylidene fluoride membranes. The membranes were then blocked with 5% skim milk in TBST and subsequently incubated with primary antibodies against *α*-SMA (Servicebio, GB111364, 1 : 800), ASK1 (Boster Bio, A00929, 1 : 1000), p-ASK1 (Cell Signaling Technology, #3765, 1 : 1000), Col I (Boster Bio, PB0981, 1 : 1000), Col III (Boster Bio, M00788, 1 : 1500), GRP78 (Boster Bio, PB0669, 1 : 1200), PERK (Boster Bio, A01992-2, 1 : 1500), p-PERK (Cell Signaling Technology, #3179, 1 : 1000), CHOP (Abcam, ab179823, 1 : 2000), CD63 (Boster Bio, PB9250, 1 : 1500), TSG101 (Boster Bio, PB0550, 1 : 1000), and GAPDH (Boster Bio, BM1623, 1 : 1000) overnight at 4°C. The next morning, the membranes were washed with TBST and then incubated with the HRP-conjugated secondary antibody for 2 h at room temperature. The immunostained protein bands were visualized by enhanced chemiluminescence, with GAPDH serving as an internal control.

### 2.7. Measurements of ROS

The levels of intracellular ROS in LX-2 cells were analyzed using an ROS assay kit according to the manufacturer's instructions. In brief, cells at a density of 1 × 10^5^ cells per well were cultured overnight in 6-well plates. The next day, the cells were incubated with 10 *μ*M 2′,7′-dichlorofluorescin diacetate (DCFH2-DA) at 37°C for 30 min. After being washed three times with PBS, the total amount of ROS was calculated based on the fluorescence intensity detected by a BD Accuri C6 flow cytometer equipped with ImageJ 1.44 software.

### 2.8. ELISA

LX-2 cells were plated in triplicate into six-well plates, after which, the corresponding cell supernatants were collected for an analysis of proinflammatory cytokine (IL-1*β*, IL-18, and TNF-*α*) levels performed by using ELISA kits provided by Jiangsu Baolai Biotechnology Co., Ltd. (Jiangsu, China). All analyses were performed according to instructions provided by the kit manufacturer.

### 2.9. Exosome Isolation and Identification

Exosomes were prepared from the cell supernatants of different groups by differential ultracentrifugation as previously described [30]. In brief, the culture medium was quickly filtered to separate intact cells and debris. Next, the cells were pelleted by ultraspeed centrifugation, resuspended in 100 mL of cold PBS, and then transferred into a low adhesive tube for storage in a -80°C refrigerator. A drop of the exosome suspension was stained with 2% uranyl acetate for 30 s and then fixed with 2% paraformaldehyde. After being allowed to dry for 60 min, the structure and size of the particles isolated from the cell culture supernatants were determined by electron microscopy.

### 2.10. Cocultures

To investigate the role played by exosomes in Ang II-induced hepatic fibrosis, quiescent HSC cells were cocultured with exosomes or activated HSC cells. After 48 h of coculture, the cells were collected for analysis of *α*-SMA expression by western blotting and immunofluorescence staining.

### 2.11. Statistical Analysis

All results represent data obtained from three independent experiments and are expressed as the mean value ± standard deviation (SD). Statistical comparisons between two groups were performed using Student's *t* test, while comparisons among more than two groups were performed using the Tukey post hoc test. A *p* value < 0.05 was considered to be statistically significant.

## 3. Results

### 3.1. Activation of HSC Cells by Ang II

**Figure 1:**
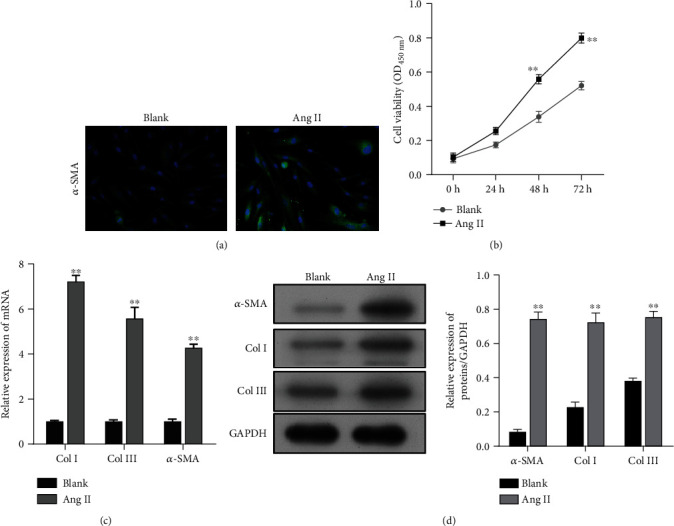
Preparation of activated HSCs induced by Ang II. (a) Immunofluorescence images of *α*-SMA in LX-2 cells with or without Ang II treatment. (b) The viability of LX-2 cells from the Ang II and blank groups was determined by the CCK-8 assay. (c) Quantitative real-time PCR and (d) western blotting were performed to measure the levels of *α*-SMA, Col I, and Col III expression in LX-2 cells with or without Ang II treatment. Data are expressed as the mean value ± standard deviation (SD). ^∗∗^*p* < 0.01, compared with the blank group.

Activation of hepatic stellate cells (HSCs) is now well established as the dominant pathogenic event that occurs during the process of liver fibrosis due to liver injury. Here, LX-2 HSCs were activated by treatment with Ang II. As shown in Figure 1(a), immunofluorescence staining revealed increased levels of *α*-SMA expression in LX-2 cells after Ang II treatment, and CCK-8 assays showed that Ang II significantly increased LX-2 cell viability when compared with the blank group (Figure 1(b)). Moreover, when compared with LX-2 cells without Ang II treatment, the levels of *α*-SMA, Col I, and Col III proteins (markers of activated HSCs), as well as the levels of mRNA for those proteins, were significantly increased in the Ang II-treated LX-2 cells (Figures 1(c) and 1(d)). Taken together, these data indicated that the LX-2 cells had been successfully activated by treatment with Ang II.

### 3.2. Expression of p-ASK1, ERS-Related Proteins, and Proinflammatory Cytokines in Activated HSC Cells

**Figure 2:**
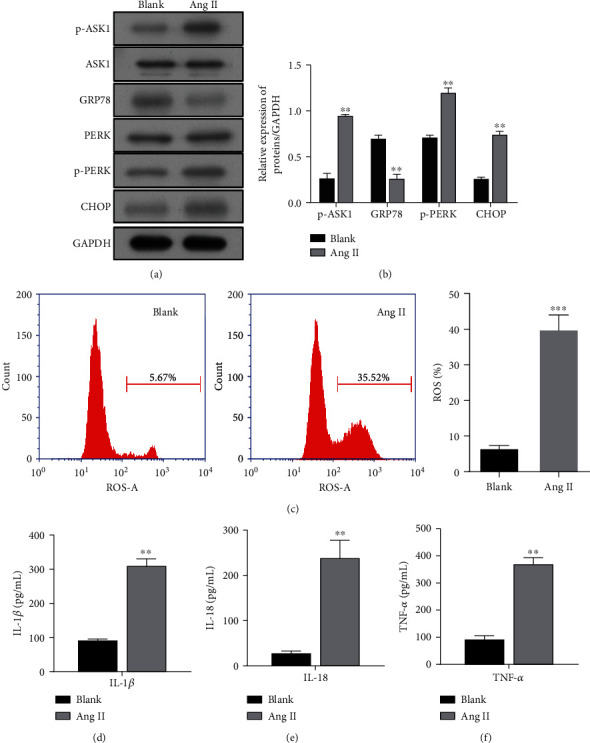
Expression of p-ASK1, ER stress-related proteins, and proinflammatory cytokines in activated HSC cells. LX-2 cells were treated with Ang II. (a) The protein bands of p-ASK1, ASK1, GRP78, PERK, p-PERK, and CHOP as well as (b) the corresponding protein levels are shown. (c) The ROS levels measured using flow cytometry are shown in the left panel, and quantified results for ROS generation are depicted in the right panel. ELISA methods were used to determine the expression levels of proinflammatory cytokines, IL-1*β* (d), IL-18 (e), and TNF-*α* (f). Results are expressed as the mean value ± standard deviation (SD). ^∗∗^*p* < 0.01 and ^∗∗∗^*p* < 0.001, compared with the blank group.

Subsequently, we found that p-ASK1 protein expression was upregulated and the levels of ERS-related proteins p-PERK and CHOP were increased, while GRP78 expression was decreased in the activated HSC cells (Figures 2(a) and 2(b)). Moreover, Ang II treatment significantly increased the intracellular ROS levels in LX-2 cells (Figure 2(c)). In addition, we assessed the effects of Ang II on the expression of proinflammatory cytokines in LX-2 cells. The results showed that the levels of proinflammatory cytokines IL-1*β* (Figure 2(d)), IL-18 (Figure 2(e)), and TNF-*α* (Figure 2(f)) were significantly increased in LX-2 cells after Ang II treatment. These data suggested that upregulation of ASK1 might be associated with Ang II-induced liver fibrosis *in vitro*.

### 3.3. ERS Caused Exosome Release from Activated HSC Cells

**Figure 3:**
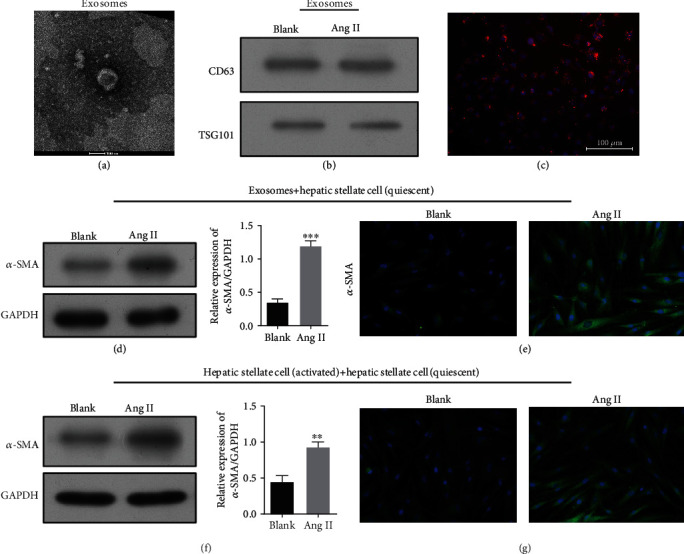
ERS caused exosome release from activated HSC cells. Exosomes released by LX-2 cells in the Ang II and blank groups were detected by electron microscopy (a). (b) Western blot analysis of *α*-SMA expression in exosomes released by LX-2 cells in the Ang II and blank groups. PKH-26-labeled exosomes (red) were taken up by LX-2 cells (c). Scale bar, 100 nm. Western blotting (d) and immunofluorescence staining (e) of *α*-SMA in quiescent HSC cells after incubated with exosomes released from activated HSC cells. Western blotting (f) and immunofluoresce nce staining (g) of *α*-SMA in quiescent HSC cells cocultured with activated HSCs. ^∗∗^*p* < 0.01 and ^∗∗∗^*p* < 0.001 vs. blank.

A related study showed that activation of ERS was correlated with the release of exosomes from liver cells [31]. Here, we isolated and purified exosomes from the supernatants of LX-2 cells in the Ang II and blank groups. The structure and size of the particles isolated from cell culture supernatants were determined by electron microscopy (Figure 3(a)). The exosome markers CD63 and TSG were detected on exosomes from the Ang II-treated LX-2 cells and the blank group by western blotting (Figure 3(b)). As shown in Figure 3(c), PKH-26-labeled exosomes (red) were taken up by LX-2 cells. Subsequently, the quiescent HSC cells were cocultured with exosomes or activated HSC cells. Western blot studies (Figure 3(d)) and immunofluorescence staining (Figure 3(e)) showed that Ang II treatment also upregulated the expression of *α*-SMA in cocultures of exosomes and quiescent HSC cells. Similar results were also found in quiescent HSC cells cocultured with activated HSCs (Figures 3(f) and 3(g)). Notably, the fold increase in the *α*-SMA expression was higher in the cocultures of exosomes and quiescent HSC cells than in quiescent HSC cells cocultured with activated HSCs indicating that the ERS-induced release of exosomes occurred from the activated HSC cells.

### 3.4. Effect of Exosomes Released by Activated HSC Cells on Quiescent HSC Cells Could Be Blocked by Annexin

**Figure 4:**
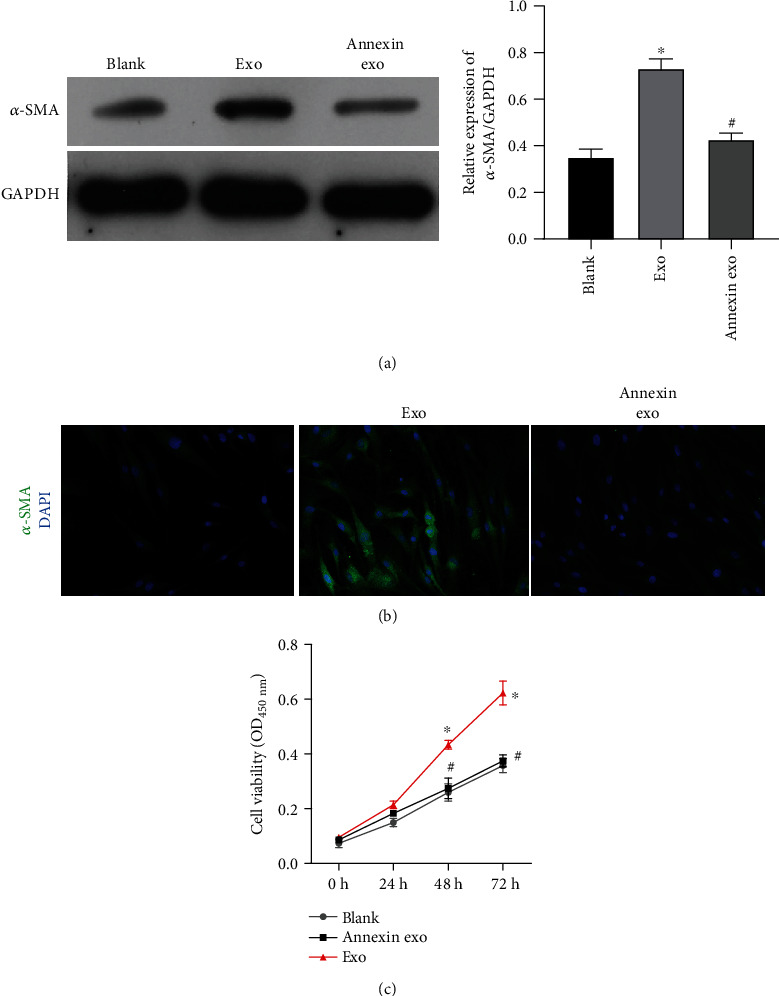
*α*-SMA expression, cell fibrogenesis, and cell viability were attenuated in HSCs treated with exosomes that had been preincubated with annexin. (a) Western blotting was used to measure *α*-SMA expression. (b) Immunofluorescence assays were performed to measure *α*-SMA expression. (c) Cell viability was determined by the CCK-8 assay. ^∗^*p* < 0.05 vs. blank; ^#^*p* < 0.05 vs. exo.

To explore the exact effect of exosomes on quiescent HSC cells, we preincubated the exosomes released by activated HSC cells with annexin, which blocked their uptake by quiescent HSC cells. As shown in Figure 4(a), the quiescent HSCs incubated with exosomes blocked by annexin displayed lower levels of *α*-SMA expression than HSCs incubated with exosomes that had not been pretreated with annexin. Subsequent studies that used immunofluorescence staining showed similar effects (Figure 4(b)). Meanwhile, HSCs incubated with the nonpretreated exosomes were significantly more viable than the HSCs treated with annexin pretreated exosomes (Figure 4(c)).

### 3.5. Effects of an ASK1 Inhibitor on Ang II-Mediated Viability and ECM-Related Protein Expression in LX-2 Cells

**Figure 5:**
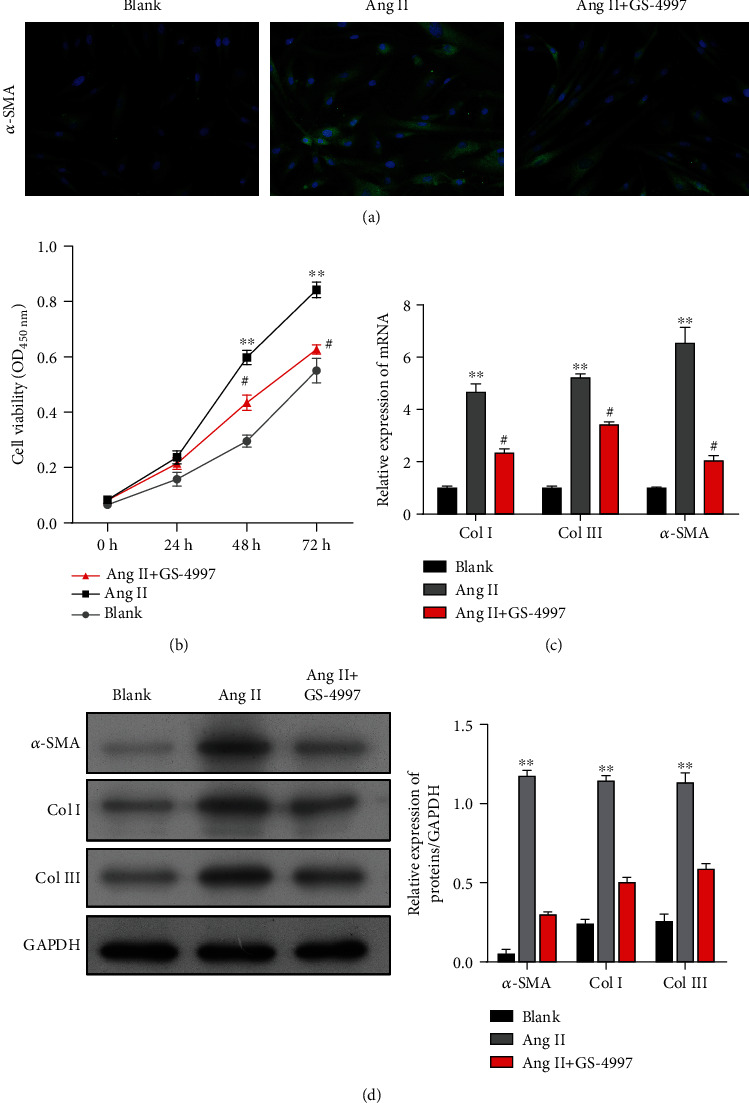
Effects of an ASK1 inhibitor on Ang II-mediated proliferation and ECM-related protein expression in LX-2 cells. LX-2 cells were treated with Ang II alone or cotreated with Ang II and an ASK1 inhibitor (GS-4997). (a) Immunofluorescence images of *α*-SMA in LX-2 cells. (b) Cell viability was determined by the CCK-8 assay. (c) Quantitative real-time PCR and (d) western blotting were performed to measure the levels of *α*-SMA, Col I, and Col III expression in LX-2 cells. Results are expressed as the mean value ± standard deviation (SD). ^∗∗^*p* < 0.01 vs. blank; ^#^*p* < 0.05 vs. Ang II.

Because a change in ASK1 phosphorylation was observed in Ang II-stimulated LX-2 cells, the specific ASK1 inhibitor GS-4997 was employed to investigate the role of ASK1 in the Ang II-mediated viability and expression of ECM-related proteins in LX-2 cells. Immunofluorescence staining showed that the increase in *α*-SMA expression caused by Ang II treatment was obviously attenuated by GS-4997 treatment (Figure 5(a)). Moreover, CCK-8 assays showed that GS-4997 could effectively suppress the viability of LX-2 cells that were induced by Ang II treatment (Figure 5(b)). A further analysis of ECM-related protein expression by quantitative real-time PCR (Figure 5(c)) and western blotting (Figure 5(d)) indicated that the upregulation of *α*-SMA, Col I, and Col III expression in LX-2 cells treated with Ang II alone was obviously less than that induced by cotreatment with Ang II plus GS-4997.

### 3.6. Effects of an ASK1 Inhibitor on Ang II-Mediated ERS and Proinflammatory Phenomena in LX-2 Cells

**Figure 6:**
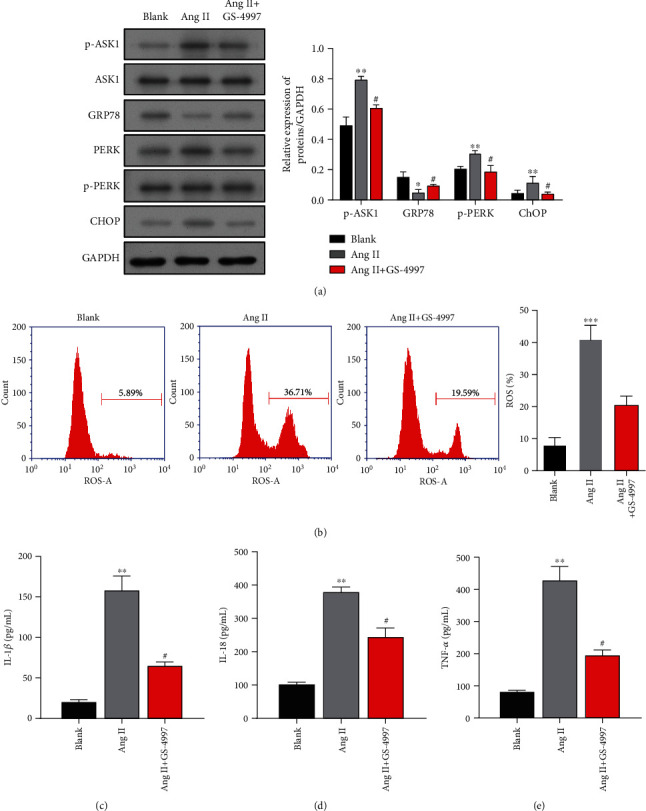
Effects of an ASK1 inhibitor on Ang II-mediated ERS and proinflammatory phenomena in LX-2 cells. LX-2 cells were treated with Ang II alone or cotreated with Ang II and an ASK1 inhibitor (GS-4997). (a) The protein bands of p-ASK1, ASK1, GRP78, PERK, p-PERK, and CHOP are shown in the left panel, and the corresponding protein levels are shown in the right panel. (b) The ROS levels measured using flow cytometry are shown in the left panel, and quantified results for ROS generation are depicted in the right panel. ELISA was used to determine the expression levels of proinflammatory cytokines, including IL-1*β* (c), IL-18 (d), and TNF-*α* (e). Results are expressed as the mean value ± standard deviation (SD). ^∗^*p* < 0.05, ^∗∗^*p* < 0.01, and ^∗∗∗^*p* < 0.001 vs. blank. ^#^*p* < 0.05 vs. Ang II.

We next sought to test whether ASK1 inhibition was involved in Ang II-mediated ERS and proinflammatory phenomena. As shown in Figure 6(a), pretreatment of LX-2 cells with the ASK1 inhibitor GS-4997 significantly blocked the expression of Ang II-induced p-ASK1, GRP78, p-PERK, and CHOP. Meanwhile, the elevated intracellular ROS levels in activated HSC cells were significantly decreased by GS-4997 pretreatment (Figure 6(b)). In addition, GS-4997 markedly reduced the enhancement effects of Ang II on IL-1*β* (Figure 6(c)), IL-18 (Figure 6(d)), and TNF-*α* expression (Figure 6(e)).

### 3.7. Effects of an ASK1 Inhibitor on ERS Caused Exosome Release by Activated HSC Cells

**Figure 7:**
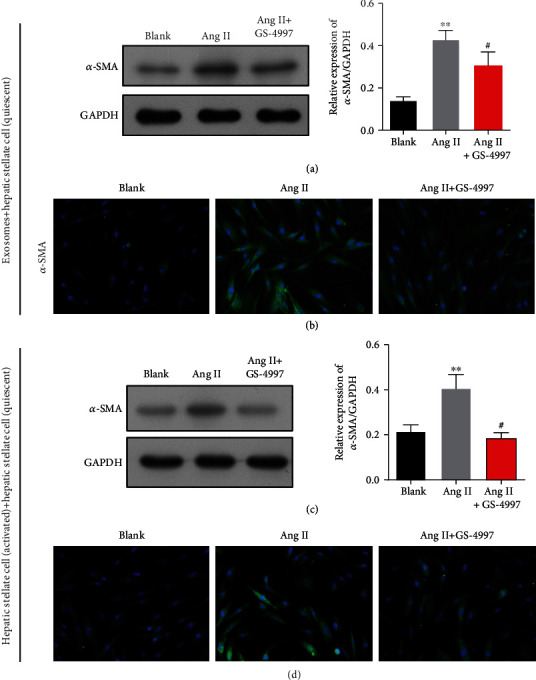
Effects of an ASK1 inhibitor on ERS-induced exosome release by activated HSC cells. LX-2 cells were treated with Ang II alone or cotreated with Ang II and an ASK1 inhibitor (GS-4997). Western blotting (a) and immunofluorescence staining (b) of *α*-SMA are shown in quiescent HSC cells incubated with activated HSCs. Western blotting (c) and immunofluorescence staining (d) of *α*-SMA are shown in quiescent HSCs cocultured with activated HSCs. Results are expressed as the mean value ± standard deviation (SD). ^∗∗^*p* < 0.01 vs. blank. ^#^*p* < 0.05 vs. Ang II.

Because an ASK1 inhibitor could suppress ERS, we explored whether altering the release of exosomes by decreasing ERS might affect the activated HSC cells. Western blot studies (Figure 7(a)) and immunofluorescence staining (Figure 7(b)) showed that the increases in *α*-SMA expression induced by Ang II treatment were obviously downregulated by GS-4997 pretreatment in quiescent HSCs after incubated with exosomes from activated HSC. Consistent with that finding, GS-4997 pretreatment remarkably attenuated the increase in *α*-SMA expression caused by Ang II treatment in quiescent HSCs after cocultured with activated HSCs (Figures 7(c) and 7(d)). These results suggest that ASK1-induced exosomes might be responsible for Ang II-induced liver fibrosis *in vitro*.

### 3.8. Knockdown of ASK1 Reversed the Effect of Ang II on HSCs

**Figure 8:**
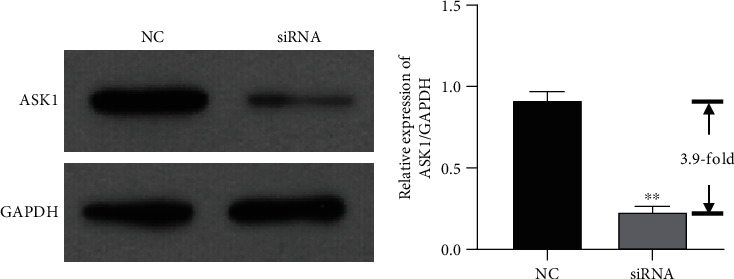
Expression of ASK1 was inhibited by siRNA. Expression of ASK1 was significantly suppressed by siRNA-targeting ASK1. ^∗∗^*p* < 0.01 vs. NC.

**Figure 9:**
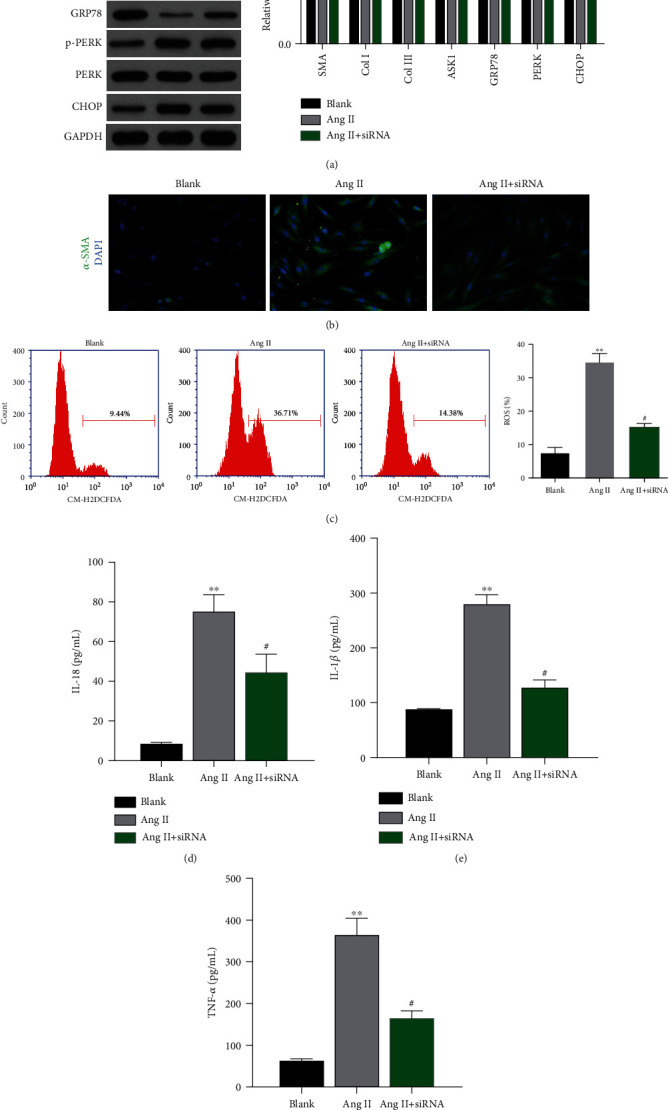
Knockdown of ASK1 attenuated ERS, ROS production, and inflammatory factors. (a) The production of fibroid- and ERS-related proteins was inhibited by ASK1 siRNA. (b) Immunofluorescence assays were performed to measure *α*-SMA expression in HSCs. (c) ROS production was inhibited by ASK1 siRNA. (d–f) The production of inflammatory factors IL-18 (d), IL-1*β* (e), and TNF-*α* (f) was blocked by ASK1 siRNA. ^∗^*p* < 0.05 and ^∗∗^*p* < 0.01 vs. blank; ^#^*p* < 0.05 vs. Ang II.

siRNA-targeting ASK1 was used to verify the effect ASK1 on HSCs. As shown in Figure 8, ASK1 expression was downregulated by siRNA-targeting ASK1. Furthermore, the expression of proteins related to activation of HSCs, such as *α*-SMA, Col I, and Col III, was elevated in cells treated with Ang II; however, those elevated expression levels were significantly attenuated by transfection with ASK1 siRNA (Figure 9(a)). Moreover, the levels of other proteins related to ERS, such as GRP78, PERK, and CHOP, were also increased by Ang II and later reduced by ASK1 siRNA transfection (Figure 9(a)). The levels of *α*-SMA expression in HSCs were also measured by immunofluorescence. Immunofluorescence assays showed levels of *α*-SMA that were similar to those in Figure 9(a). Next, ROS production and inflammatory factors were measured to investigate the stress response in HSCs. As shown in Figure 9(b), transfection with ASK1 siRNA had an inhibitory effect on Ang II-induced ROS production (Figure 9(c)). The levels of inflammatory factors such as IL-18, IL-1*β*, and TNF-*α* were significantly increased in Ang II-treated cells and attenuated by ASK1 siRNA (Figures 9(d)–9(f)).

### 3.9. Exosomes Released from ASK1 Knockdown HSCs Reversed the Effect of *α*-SMA Expression

**Figure 10:**
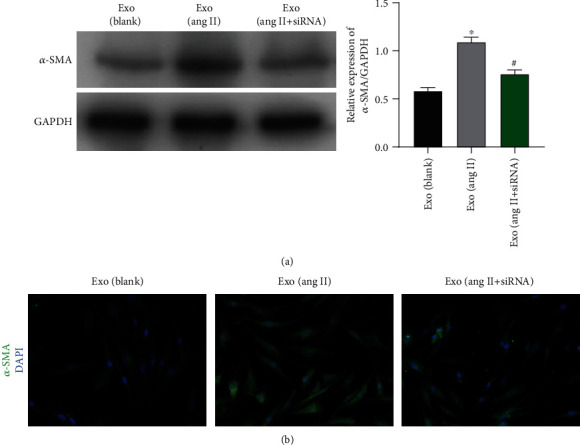
Exosomes from HSCs transfected with ASK1 siRNA showed lower levels of *α*-SMA expression. Western blot (a) and immunofluorescence assays (b) were used to measure *α*-SMA expression in HSCs that had been incubated with exosomes from blank HSCs, Ang II-treated HSCs, and Ang II+siRNA-treated HSCs, respectively. ^∗^*p* < 0.05 vs. blank; ^#^*p* < 0.05 vs. Ang II.

To explore the effect of exosomes released from activated HSCs transfected with siRNA, such exosomes were isolated and incubated with quiescent HSCs. As shown in Figure 10(a), *α*-SMA expression was increased when the quiescent HSCs were incubated with exosomes from Ang II-treated cells. However, the levels of *α*-SMA expression in HSCs were lower when the HSCs were treated with ASK1 siRNA rather than EXO (Ang II). Those trends in *α*-SMA expression were further validated by immunofluorescence assays (Figure 10(b)).

## 4. Discussion

In this study, we first demonstrated that ASK1 mediates the Ang II-induced activation of HSCs and thereby liver fibrosis *in vitro*. The principal findings obtained in our study are as follows: (1) the expression of phosphorylated ASK1 was upregulated in Ang II-induced liver fibrosis and under conditions of ERS *in vitro*, (2) an ASK1 inhibitor (GS-4997) significantly reversed Ang II-induced liver fibrosis and ERS *in vitro*, and (3) ASK1-mediated ERS-induced exosome release was involved in Ang II-induced liver fibrosis *in vitro*.

In the first part of our study, we successfully employed Ang II stimulation to establish an *in vitro* model of liver fibrosis in LX-2 cells, as reflected by increased cell viability, inflammation, ERS, ROS, and ECM-related proteins (*α*-SMA, Col I, and Col III) in the Ang II-treated LX-2 cells. This was in line with a previous finding that Ang II could stimulate HSC activation in myocardial fibrosis, as well as increases in cell viability , *α*-SMA, and Col I expression and increased levels of ERK and phosphorylated c-Jun [20]. Previous studies have shown that a higher angiotensin level is a common underlying factor that contributes to ERS [32–34]. In alveolar epithelial cells, ERS-induced apoptosis is mediated by the autocrine Ang II/Ang I-VII system [35]. Moreover, infusion of Ang II was shown to enhance the activation of epidermal growth factor receptors and ERS in mediation of cardiovascular hypertrophy and perivascular fibrosis [36. Evidence also indicated that Ang II can induce hepatic fibrosis in rats by stimulating NF-*κ*B and AP1 activation [14]. Other reports suggest that NLPR3 is involved in promoting hepatic fibrosis [37, 38]. This evidence led us to conclude that Ang II signaling plays a crucial role in the pathogenesis of fibrosis.

ERS and inflammation response has been widely recognized in hepatic fibrosis [39, 40]. As a matter of fact, researchers notice that an increase of ER stress response can result in activation of NF-*κ*B [41, 42]. Also, JNK has been regarded as a factor that connects ERS to inflammation [42]. Zhao et al. suggests that Gambogenic acid mediates ERS in colorectal cancer cell through the IRE1*α*/JNK pathway [43]. A previous study suggests that ERS has been evoked in the LPS-treated mouse granulosa cell; interestingly, ERS has been blocked by the TLR4 inhibitor TAK-242 [44]. Therefore, there is a complex relationship between ERS and inflammation response. Duvigneau et al. has concluded that there might be a feedback loop between ERS and inflammation response in liver diseases [45]. In this study, inflammation was enhanced by Ang II treatment in LX-2 cells, with the ASK1 expression and ERS response elevated, while it was reversed by the ASK1 inhibitor or siRNA-targeting ASK1. Meanwhile, inflammation response was also inhibited. Further investigation needs to be focused on which, either ERS or inflammation response, should be the switch to another.

Potential relationships between ASK1 and fibrosis have been reported in the following studies: Liles et al. [46] showed that ASK1 activation was a central mediator of kidney fibrosis and dysfunction and that ASK1 inhibition is a promising approach for reducing the deleterious downstream consequences of oxidative stress in the kidney. Amos et al. [47] demonstrated that an ASK1 inhibitor (GS-444217) reduced kidney inflammation and fibrosis by suppressing p38 MAPK/JNK signaling. Furthermore, pretreatment with ASK1 inhibitors (G2261818A and G2358939A) was shown to dose-dependently attenuate Ang II-stimulated neonatal rat cardiac myocyte (NCM) hypertrophy and hypertrophic gene expression, as well as neonatal rat cardiac fibroblast collagen synthesis and profibrotic gene expression [48]. Consistent with those findings, our *in vitro* data showed that an ASK1 inhibitor (GS-4997) suppressed Ang II-mediated viability, ECM-related protein expression, ERS, and proinflammatory phenomena in HSCs. GS-4997 is a key mediator of the deleterious effects of oxidative stress and has been evaluated in a phase 2 clinical trial conducted in patients with diabetic kidney disease [49].

In addition to activated ERS, liver fibrosis is associated with exosome cargo delivery and various exosome-regulated functions in HSCs [50]. Here, we found that ASK1-mediated ERS caused the release of exosomes by Ang II-activated HSCs. An association between ERS and exosome release has been previously reported as follows: (1) mesenchymal stem cell-exosomes were shown to attenuate ERS-induced apoptosis by activating AKT and ERK signaling to ameliorate intervertebral disc degeneration [51]; (2) platelet-rich plasma-exosomes were shown to prevent glucocorticoid-induced apoptosis in a rat model of osteonecrosis of the femoral head under conditions of ERS [52]. Studies have shown that exosomes released from hepatic stellate cells are involved in the functional regulation of liver disease. Qu et al. [53] suggested that exosomes released from adipose-derived mesenchymal stem cells carry miR-181-5p and transfer miR-181-5p into damaged liver cells to exert an antifibrotic effect. A recent report revealed that multivesicular body- (MVB-) derived exosomes created by autophagy promote fibrosis in HSCs via the mTOR signaling pathway [54]. Exosomes from HSCs can carry proteins such as histones and keratins, which contribute to HSC functions *in vivo* [55]. Our data led us to conclude that exosomes from already activated HSCs subsequently activated quiescent HSCs and thereby served as fibrotic factors. However, in this study, we did not investigate the contents of the exosomes derived from Ang II-activated HSCs. Also, future investigations should focus on the *in vivo* effects of exosomes derived from activated HSCs.

## 5. Conclusion

**Figure 11:**
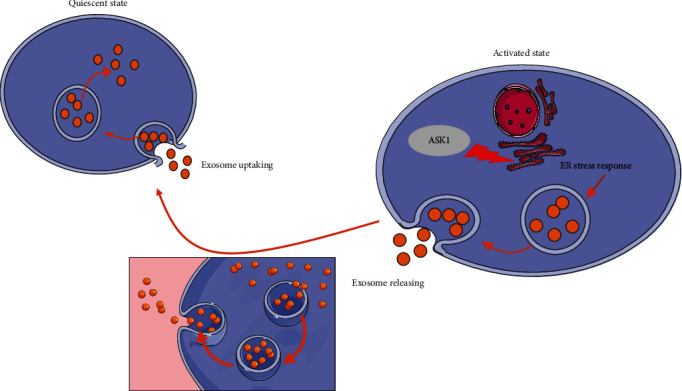
Schematic presentation of this study. An ERS response was evoked by ASK1 activation, which caused Ang II-induced HSCs to release exosomes. The exosomes released by activated HSCs were taken up by quiescent HSCs, which were then activated by the exosomes.

In conclusion, we identified Ang II-induced ASK1 as a crucial factor that initiates HSC activation by promoting ERS. We further demonstrated that GS-4997, a selective small-molecular inhibitor of ASK1, could significantly reverse Ang II-induced liver fibrosis *in vitro* (Figure 11). Taken together, our data suggest that downregulation of ASK1 might be a promising strategy for treating liver fibrosis. The specific contents of exosomes and the role of exosomes *in vivo* should be further explored.

## Figures and Tables

**Table 1:** Primers' sequences used in qRT-PCR in this study.

ID	Sequence (5′-3′)	Product length (bp)
GAPDH F	CCTCGTCTCATAGACAAGATGGT	169
GAPDH R	GGGTAGAGTCATACTGGAACATG	
Col I 2F	GATCGACCCTAACCAAGGCT	113
Col I 2R	ACCAGTTCTTCTGAGGCACA	
*α*-SMA F	GGGCCAAAAGGACAGCTATG	86
*α*-SMA R	TGATGCCGTGTTCTATCGGA	
Col III F	ATTCCTGGGAGAAATGGCGA	154
Col III R	TTCCTCCGACTCCAGACTTG	

F: forward primer; R: reversed primer.

## Data Availability

The data used to support the findings of this study are included within the article.

## References

[1] Popov Y., Schuppan D. (2009). Targeting liver fibrosis: strategies for development and validation of antifibrotic therapies. *Hepatology*.

[2] Tsuchida T., Friedman S. L. (2017). Mechanisms of hepatic stellate cell activation. *Nature Reviews. Gastroenterology & Hepatology*.

[3] Puche J. E., Saiman Y., Friedman S. L. (2013). Hepatic stellate cells and liver fibrosis. *Comprehensive Physiology*.

[4] Sun M., Kisseleva T. (2015). Reversibility of liver fibrosis. *Clinics and Research in Hepatology and Gastroenterology*.

[5] Stahl P. D., Raposo G. (2018). Exosomes and extracellular vesicles: the path forward. *Essays in Biochemistry*.

[6] Shen J., Huang C. K., Yu H. (2017). The role of exosomes in hepatitis, liver cirrhosis and hepatocellular carcinoma. *Journal of Cellular and Molecular Medicine*.

[7] Lou G., Chen Z., Zheng M., Liu Y. (2017). Mesenchymal stem cell-derived exosomes as a new therapeutic strategy for liver diseases. *Experimental & Molecular Medicine*.

[8] Rong X., Liu J., Yao X., Jiang T., Wang Y., Xie F. (2019). Human bone marrow mesenchymal stem cells-derived exosomes alleviate liver fibrosis through the Wnt/*β*-catenin pathway. *Stem Cell Research & Therapy*.

[9] Wan L., Xia T., du Y. (2019). Exosomes from activated hepatic stellate cells contain GLUT1 and PKM2: a role for exosomes in metabolic switch of liver nonparenchymal cells. *The FASEB Journal*.

[10] Paizis G., Gilbert R. E., Cooper M. E. (2001). Effect of angiotensin II type 1 receptor blockade on experimental hepatic fibrogenesis. *Journal of Hepatology*.

[11] Yoshiji H. (2001). Angiotensin-II type 1 receptor interaction is a major regulator for liver fibrosis development in rats. *Hepatology*.

[12] Bataller R., Ginès P., Nicolás J. M. (2000). Angiotensin II induces contraction and proliferation of human hepatic stellate cells. *Gastroenterology*.

[13] Bataller R., Gäbele E., Schoonhoven R. (2003). Prolonged infusion of angiotensin II into normal rats induces stellate cell activation and proinflammatory events in liver. *American Journal of Physiology. Gastrointestinal and Liver Physiology*.

[14] Li X., Meng Y., Wu P., Zhang Z., Yang X. (2007). Angiotensin II and aldosterone stimulating NF-kappaB and AP-1 activation in hepatic fibrosis of rat. *Regulatory Peptides*.

[15] Jin H., Yamamoto N., Uchida K., Terai S., Sakaida I. (2007). Telmisartan prevents hepatic fibrosis and enzyme-altered lesions in liver cirrhosis rat induced by a choline-deficient L-amino acid-defined diet. *Biochemical and Biophysical Research Communications*.

[16] Yu F. J., Dong P. H., Fan X. F., Lin Z., Chen Y. P., Li J. (2010). Down-regulation of angiotensin II by shRNA reduces collagen synthesis in hepatic stellate cells. *International Journal of Molecular Medicine*.

[17] Hirose A., Ono M., Saibara T. (2007). Angiotensin II type 1 receptor blocker inhibits fibrosis in rat nonalcoholic steatohepatitis. *Hepatology*.

[18] Nabeshima Y., Tazuma S., Kanno K. (2006). Anti-fibrogenic function of angiotensin II type 2 receptor in CCl4-induced liver fibrosis. *Biochemical and Biophysical Research Communications*.

[19] Rockey D. C. (2008). Current and future anti-fibrotic therapies for chronic liver disease. *Clinics in Liver Disease*.

[20] Li S., Wang L., Yan X. (2012). Salvianolic acid B attenuates rat hepatic fibrosis via downregulating angiotensin II signaling. *Evidence-based Complementary and Alternative Medicine*.

[21] Yang C., Wang Y., Liu H. (2012). Ghrelin protects H9c2 cardiomyocytes from angiotensin II-induced apoptosis through the endoplasmic reticulum stress pathway. *Journal of Cardiovascular Pharmacology*.

[22] Hsieh C. C., Papaconstantinou J. (2005). Thioredoxin-ASK1 complex levels regulate ROS-mediated p38 MAPK pathway activity in livers of aged and long-lived Snell dwarf mice. *The FASEB Journal*.

[23] Ichijo H., Nishida E., Irie K. (1997). Induction of apoptosis by ASK1, a mammalian MAPKKK that activates SAPK/JNK and p38 signaling pathways. *Science*.

[24] Pan J., Chang Q., Wang X. (2010). Reactive oxygen species-activated Akt/ASK1/p38 signaling pathway in nickel compound-induced apoptosis in BEAS 2B cells. *Chemical Research in Toxicology*.

[25] Nakagawa H., Hirata Y., Takeda K. (2011). Apoptosis signal-regulating kinase 1 inhibits hepatocarcinogenesis by controlling the tumor-suppressing function of stress-activated mitogen-activated protein kinase. *Hepatology*.

[26] Sui X., Kong N., Ye L. (2014). p38 and JNK MAPK pathways control the balance of apoptosis and autophagy in response to chemotherapeutic agents. *Cancer Letters*.

[27] Yamada S., Noguchi H., Tanimoto A. (2017). Critical and diverse in vivo roles of apoptosis signal-regulating kinase 1 in animal models of atherosclerosis and cholestatic liver injury. *Histology and Histopathology*.

[28] Noguchi H., Yamada S., Nabeshima A. (2014). Depletion of apoptosis signal-regulating kinase 1 prevents bile duct ligation-induced necroinflammation and subsequent peribiliary fibrosis. *The American Journal of Pathology*.

[29] Livak K. J., Schmittgen T. D. (2001). Analysis of relative gene expression data using real-time quantitative PCR and the 2(-delta delta C(T)) method. *Methods*.

[30] Li H., Chi X., Li R., Ouyang J., Chen Y. (2019). HIV-1-infected cell-derived exosomes promote the growth and progression of cervical cancer. *International Journal of Biological Sciences*.

[31] Liu J., Fan L., Yu H. (2019). Endoplasmic reticulum stress causes liver cancer cells to release exosomal miR-23a-3p and up-regulate programmed death ligand 1 expression in macrophages. *Hepatology*.

[32] Chan S. M. H., Lau Y.-S., Miller A. A. (2017). Angiotensin II causes *β*-cell dysfunction through an ER stress-induced proinflammatory response. *Endocrinology*.

[33] Szegezdi E., Logue S. E., Gorman A. M., Samali A. (2006). Mediators of endoplasmic reticulum stress-induced apoptosis. *EMBO Reports*.

[34] Xie G., Liu Y., Yao Q. (2017). Hypoxia-induced angiotensin II by the lactate-chymase-dependent mechanism mediates radioresistance of hypoxic tumor cells. *Scientific Reports*.

[35] Uhal B. D., Nguyen H., Dang M. T. (2013). Abrogation of ER stress-induced apoptosis of alveolar epithelial cells by angiotensin 1-7. *American Journal of Physiology. Lung Cellular and Molecular Physiology*.

[36] Takayanagi T., Forrester S. J., Kawai T. (2016). Vascular ADAM17 as a novel therapeutic target in mediating cardiovascular hypertrophy and perivascular fibrosis induced by angiotensin II. *Hypertension*.

[37] Ning Z. W., Luo X. Y., Wang G. Z. (2017). MicroRNA-21 mediates angiotensin II-induced liver fibrosis by activating NLRP3 inflammasome/IL-1*β* axis via targeting Smad7 and Spry1. *Antioxidants & Redox Signaling*.

[38] Cai S. M., Yang R. Q., Li Y. (2016). Angiotensin-(1-7) improves liver fibrosis by regulating the NLRP3 inflammasome via redox balance modulation. *Antioxidants & Redox Signaling*.

[39] Hu N., Guo C., Dai X. (2020). Forsythiae Fructuse water extract attenuates liver fibrosis via TLR4/MyD88/NF-*κ*B and TGF-*β*/smads signaling pathways. *Journal of Ethnopharmacology*.

[40] Maiers J. L., Malhi H. (2019). Endoplasmic reticulum stress in metabolic liver diseases and hepatic fibrosis. *Seminars in Liver Disease*.

[41] Jiang M., Wang H., Liu Z. (2020). Endoplasmic reticulum stress-dependent activation of iNOS/NO-NF-*κ*B signaling and NLRP3 inflammasome contributes to endothelial inflammation and apoptosis associated with microgravity. *The FASEB Journal*.

[42] Zhang K., Kaufman R. J. (2008). From endoplasmic-reticulum stress to the inflammatory response. *Nature*.

[43] Zhao Q., Zhong J., Bi Y. (2020). Gambogenic acid induces Noxa-mediated apoptosis in colorectal cancer through ROS-dependent activation of IRE1*α*/JNK. *Phytomedicine*.

[44] LEI L., Junbang G. E., ZHAO H., WANG X., YANG L. (2019). Role of endoplasmic reticulum stress in lipopolysaccharide-inhibited mouse granulosa cell estradiol production. *The Journal of Reproduction and Development*.

[45] Duvigneau J. C., Luís A., Gorman A. M. (2019). Crosstalk between inflammatory mediators and endoplasmic reticulum stress in liver diseases. *Cytokine*.

[46] Liles J. T., Corkey B. K., Notte G. T. (2018). ASK1 contributes to fibrosis and dysfunction in models of kidney disease. *The Journal of Clinical Investigation*.

[47] Amos L. A., Ma F. Y., Tesch G. H. (2018). ASK1 inhibitor treatment suppresses p38/JNK signalling with reduced kidney inflammation and fibrosis in rat crescentic glomerulonephritis. *Journal of Cellular and Molecular Medicine*.

[48] Yang W., Wang B. H., Wang I. (2019). Inhibition of apoptosis signal-regulating kinase 1 attenuates myocyte hypertrophy and fibroblast collagen synthesis. *Heart, Lung & Circulation*.

[49] Lin J. H., Zhang J. J., Lin S. L., Chertow G. M. (2015). Design of a phase 2 clinical trial of an ASK1 inhibitor, GS-4997, in patients with diabetic kidney disease. *Nephron*.

[50] Chen L., Brigstock D. R. (2016). Integrins and heparan sulfate proteoglycans on hepatic stellate cells (HSC) are novel receptors for HSC-derived exosomes. *FEBS Letters*.

[51] Liao Z., Luo R., Li G. (2019). Exosomes from mesenchymal stem cells modulate endoplasmic reticulum stress to protect against nucleus pulposus cell death and ameliorate intervertebral disc degeneration in vivo. *Theranostics*.

[52] Tao S. C., Yuan T., Rui B. Y., Zhu Z. Z., Guo S. C., Zhang C. Q. (2017). Exosomes derived from human platelet-rich plasma prevent apoptosis induced by glucocorticoid-associated endoplasmic reticulum stress in rat osteonecrosis of the femoral head via the Akt/Bad/Bcl-2 signal pathway. *Theranostics*.

[53] Qu Y., Zhang Q., Cai X. (2017). Exosomes derived from miR-181-5p-modified adipose-derived mesenchymal stem cells prevent liver fibrosis via autophagy activation. *Journal of Cellular and Molecular Medicine*.

[54] Gao J., Wei B., de Assuncao T. M. (2020). Hepatic stellate cell autophagy inhibits extracellular vesicle release to attenuate liver fibrosis. *Journal of Hepatology*.

[55] Li X., Chen R., Kemper S., Brigstock D. R. (2020). Dynamic changes in function and proteomic composition of extracellular vesicles from hepatic stellate cells during cellular activation. *Cell*.

